# Identification and Characterization of the *Efbzip* Gene Family in *Erianthus fulvus* and Exploration of Functional Genes Involved in Sucrose Metabolism

**DOI:** 10.3390/genes16121434

**Published:** 2025-12-01

**Authors:** Changzu Zhao, Weiyou Nong, Zhenfeng Qian, Qian Ding, Yujie Wang, Lilian He, Fusheng Li

**Affiliations:** 1 College of Agronomy and Biotechnology, Yunnan Agricultural University, Kunming 650201, China; zhaochangzu123@163.com (C.Z.); nwy1022@sina.com (W.N.); qzf19960621@sina.com (Z.Q.); 15752764954@163.com (Q.D.); 18272677633@163.com (Y.W.); 2 Sugarcane Research Institute, Yunnan Agricultural University, Kunming 650201, China; 3 The Key Laboratory for Crop Production and Smart Agriculture of Yunnan Province, Kunming 650201, China

**Keywords:** *Erianthus fulvus*, sugarcane, sucrose metabolism, *Efbzip* gene family, bZIP transcription factors

## Abstract

**Background/Objectives**: Sugarcane (*Saccharum* spp.) is a major global sugar crop, and improving sucrose accumulation is critical for industry and bioenergy. Due to its high Brix content, *Erianthus fulvus* (*E. fulvus*) is valuable for genetic improvement of sugarcane. The bZIP transcription factor family critically regulates plant sucrose metabolism, but its roles in sugarcane remain largely unexplored. **Methods**: Through bioinformatics methods, *Efbzip* gene family members were systematically identified within the genome of *E. fulvus*. Gene expression patterns in distinct plant tissues were examined by RNA-seq and quantitative real-time PCR (qRT-PCR). Furthermore, genes potentially involved in sucrose metabolism were screened using transient expression assays and subcellular localization studies conducted in tobacco. **Results**: Seventy-nine *Efbzip* genes were identified and classified into nine subgroups, showing uneven distribution across ten chromosomes. Among ten conserved motifs, Motif1 was most conserved. Subcellular localization and physicochemical analyses showed most *Efbzip* proteins were hydrophilic and nuclear-localized. Cis-regulatory element analysis suggested *Efbzip* proteins regulate sucrose metabolism through hormone and light-responsive pathways. Segmental duplication primarily drove *Efbzip* gene family expansion. qRT-PCR showed predominant expression in stems and leaves, with subgroup-specific patterns. Nuclear localization of *Efbzip52* was confirmed. Transient overexpression of *Efbzip52*, *Efbzip61*, and *Efbzip64* significantly increased sucrose content in tobacco leaves, with highly statistically significant (*p* < 0.0001). **Conclusions**: In this study, the *Efbzip* gene family of *E. fulvus* was systematically characterized for the first time. Key candidate genes potentially involved in sucrose metabolism were identified, providing potential targets for the genetic improvement of sugarcane.

## 1. Introduction

The bZIP transcription factor family has a wide distribution among eukaryotes [1,2,3]. The domains of these proteins are essentially the basic region and the leucine zipper motif. The basic region of the transcription factor recognizes the specific target DNA, and the leucine zipper is involved in dimerization, thus having the ability to bind the target gene’s DNA efficiently [4,5]. bZIP transcription factors are divided into various subfamilies, such as the A, B, C, and D subfamilies, depending on the characteristics and evolutionary distances of the proteins [6]. Previous studies have identified the *bZIP* gene family in various species, such as rice and sugarcane [7,8,9,10,11,12,13,14,15]. Functionally, bZIP transcription factors participate broadly in biological processes, including developmental regulation [16], hormonal signaling pathways [17], sucrose metabolism [18], and plant responses to abiotic stresses (e.g., salt, drought, cold, heat) [19,20,21,22] as well as biotic stresses [4,23].

Critically, bZIP transcription factors are central to regulating sugar signaling responses and metabolic gene transcription. Sugar-dependent translational regulation inhibits transcription factors such as AtbZIP11/ATB2 [18]. Enhanced sugar accumulation, including sucrose, glucose, and fructose, results from the expression of *SlbZIP1* and *SlbZIP2* gene variants lacking upstream open reading frames (uORFs), specifically during tomato fruit development [24]. In apple, phosphorylated *bZIP39* activates sorbitol metabolism by interacting with the promoter regions of the sorbitol dehydrogenase 1 (SDH1) and aldose-6-phosphate reductase (A6PR) genes [25]. The expression of MtATB2 also varies with the level of sucrose in the root nodules of legumes and corresponds with changes in root growth and senescence [26]. Overexpression of TBZ17 in tobacco leads to a significant increase in leaf sucrose content [27], while PpybZIP43 promotes sucrose synthesis in pear by inducing PpySPS3 expression [28].

Sugarcane is a worldwide sugar crop. Increasing the concentration of sugars in sugarcane is very important not only for the yield of the plant but also for the food and energy sectors. Contemporary research on sugar metabolism demonstrates that sugars serve not only as energy storage molecules but also as significant regulators of plant growth and stress response mechanisms at the cellular level [29,30,31,32]. Consequently, developing sugarcane varieties with high sugar content has become a fundamental breeding objective [33,34,35]. *E. fulvus*, a wild type of sugarcane, belongs to the Saccharinae subfamily within the Poaceae family and is notably the only diploid species in the Saccharum Complex. This type of grass is found in tropical, subtropical, and temperate climates and grows in the valleys of mountains with elevations of 1300–2400 m above sea level [36]. The beneficial characteristics of *E. fulvus* found through former research studies include its high sugar (high Brix), resistance to drought, resistance to poor soil conditions, and cold resistance [37,38,39,40]. The search for genes linked to the high-sugar trait of *E. fulvus* could be a good approach for improving the sugar level of a sugarcane plant.

Due to the complexity of the sugarcane genome [41], systematic identification of bZIP transcription factors in sugarcane remains unreported. Given the potential of *E. fulvus* for genetic improvement in sugarcane breeding programs and the significant role of bZIP transcription factors in sucrose metabolism, this study aimed to systematically characterize the *Efbzip* gene family. This research further explored candidate genes linked to sucrose metabolism, thereby addressing existing gaps in the understanding of bZIP transcription factors in *E. fulvus*.

## 2. Materials and Methods

### 2.1. Plant Materials

The ZM584 clonal line of *E. fulvus* (2n = 20, genome size 902 Mb) was obtained and preserved by the Sugarcane Research Institute of Yunnan Agricultural University. Plants were grown in pots under controlled greenhouse conditions at the same institute. This genotype reaches a Brix value of 18.6% at maturity, representing one of the high-sugar wild germplasms. In September 2025, mature roots, stems, and leaves were harvested, rapidly frozen in liquid nitrogen, and stored at −80 °C for RNA isolation and subsequent cDNA synthesis. These tissues were used for gene cloning and qRT-PCR analyses. Nicotiana benthamiana plants maintained in our laboratory were employed for subcellular localization assays and transient expression studies.

### 2.2. Identification of Gene Family and Physicochemical Analysis of Proteins

Genome data for *E. fulvus* (YN2009-3) were retrieved from the Sugarcane Genome Database [42]. *Efbzip* family members were identified using TBtools (V1.116) based on the hidden Markov model PF00170 obtained from InterPro [43]. Conserved domains of putative genes were verified through CDD search and Pfam. Physiochemical characteristics of the encoded proteins were evaluated using ExPasy (http://web.expasy.org/protparam/, accessed on 28 November 2025), and subcellular localization was inferred with the WoLF PSORT tool.

### 2.3. Phylogenetic Analysis of Efbzip Protein Family

The phylogenetic relationships among Efbzip proteins were assessed using Clustal X2, and the resulting tree was visualized with ChiPlot. The Neighbor-Joining method was applied with default settings, including a bootstrap analysis of 1000 replicates.

### 2.4. Promoter Cis-Acting Element Analysis of Efbzip Genes

The promoter regions, comprising 2000 bp upstream sequences from each *Efbzip* gene, were extracted from the *E. fulvus* genome utilizing TBtools (V1.116). The PlantCARE database was employed to predict cis-regulatory elements under default parameters, and the annotated results were subsequently visualized using TBtools.

### 2.5. Analysis of Gene Structure, Conserved Protein Motifs, and Domains in Efbzip Family Members

Gene structural analysis and conserved protein motifs were determined using TBtools (V1.116). The parameters for motif identification included a motif count of 10, minimum motif width of 6, and maximum motif width of 50, with other settings maintained at default. Conserved protein domains were identified using the CDD search tool, and all results related to gene structures, motifs, and domains were visualized through TBtools (V1.116).

### 2.6. Synteny Analysis of Efbzip Genes

Synteny analyses, both within *E. fulvus* for the *Efbzip* gene family and between *E. fulvus* and species such as *Arabidopsis thaliana*, *Saccharum spontaneum*, *Erianthus rockii*, and the sugarcane cultivar XTT22, were conducted utilizing the MCScanX plugin within the TBtools (V1.116) software (E values ≤ 1 × 10^−10^, with all other parameters set to their default values). Visualization of these synteny relationships was also accomplished through TBtools.

### 2.7. Expression Pattern Analysis of Efbzip Genes in Different Tissues

To verify expression patterns of representative *Efbzip* genes, those exhibiting elevated expression levels within each subfamily were initially selected based on transcriptome data previously reported [42]. Expression validation was conducted using quantitative real-time PCR (qRT-PCR), with experiments performed in triplicate. Statistical analysis was completed by one-way ANOVA, followed by Tukey’s multiple-comparison test, to assess significant differences among the groups.

### 2.8. RNA Extraction, cDNA Synthesis, Gene Cloning, Vector Construction, and qRT-PCR Assays

TRIzol reagent (Tiangen, Beijing, China) was employed to extract total RNA, and complementary DNA (cDNA) synthesis was performed from this RNA using the FastQuant RT Super Mix Kit (Tiangen, Beijing, China). Primers required for cloning and qRT-PCR were generated using Primer 5 software (Table S6), while those designed for homologous recombination were prepared using SnapGene 3.2.1 software (Table S3). Gene amplification was executed with PrimeSTAR Max DNA Polymerase (Takara, Beijing, China), whereas homologous recombination procedures utilized the In-Fusion Snap Assembly Master Mix (Takara, Beijing, China). qRT-PCR reactions were carried out on the ABI 7500 Real-Time PCR System using SuperReal PreMix Plus (SYBR Green) reagent (Tiangen, Beijing, China). The housekeeping gene, 25S rRNA, was employed as an internal control, and relative gene expression was calculated using the 2^−ΔΔCT^ method.

### 2.9. Subcellular Localization Assay

The pCAMBIA1300-*Efbzip*52-eGFP subcellular localization vector was constructed using homologous recombination. The vector was sequentially transformed into *Escherichia coli* and *Agrobacterium tumefaciens* competent cells. It was subsequently injected into *Nicotiana benthamiana* leaves for transient expression. Fluorescence signals were detected 72 h after injection using an Olympus FV3000 laser scanning confocal microscope (Olympus, Tokyo, Japan). Each treatment was performed in triplicate.

### 2.10. Identification of Efbzip Family Members Regulating Sucrose Metabolism

Previous studies suggest that structurally similar genes often share analogous functions. To identify potential *Efbzip* family members involved in sucrose metabolism, a phylogenetic tree was constructed using known bzip genes (*Ppybzip43*, *TBZ17*, *Atbzip11*) regulating sucrose metabolism in other species as references. Four *E. fulvus* genes (*Efbzip13*, *Efbzip52*, *Efbzip61*, and *Efbzip64*) clustered closely with these reference genes. These genes were cloned, and four overexpression vectors (pCAMBIA3301-Ubi-*Efbzip*13, pCAMBIA3301-Ubi-*Efbzip*52, pCAMBIA3301-Ubi-*Efbzip*61, and pCAMBIA3301-Ubi-*Efbzip*64) were constructed by homologous recombination using KpnI and BamHI restriction sites. For transient expression analysis, vectors were introduced into Nicotiana benthamiana plants, initially kept under dark conditions for 24 h before subsequent light exposure. Leaf tissues were harvested at two time points, 24 h (control) and 72 h post-infiltration, for both qRT-PCR assessment and sucrose quantification. Sucrose concentrations were determined using the Plant Sucrose Content Assay Kit (BOXBIO, Beijing, China). Agrobacterium tumefaciens culture with an OD_600_ value between 0.4 and 0.5 was used for the transformation experiments. Healthy Nicotiana benthamiana plants aged 6 weeks (with 4–6 true leaves) were used, with three biological replicates performed for each treatment. Two-way ANOVA was performed, followed by Tukey’s multiple-comparisons test, to determine significant differences between groups.

### 2.11. Data Analysis

All statistical analyses and graphical representations were produced using Microsoft Excel 2010 and GraphPad Prism 8.0.1 software.

## 3. Results

### 3.1. Identification of Efbzip Gene Family Members and Their Physicochemical Properties

**Figure 1:**
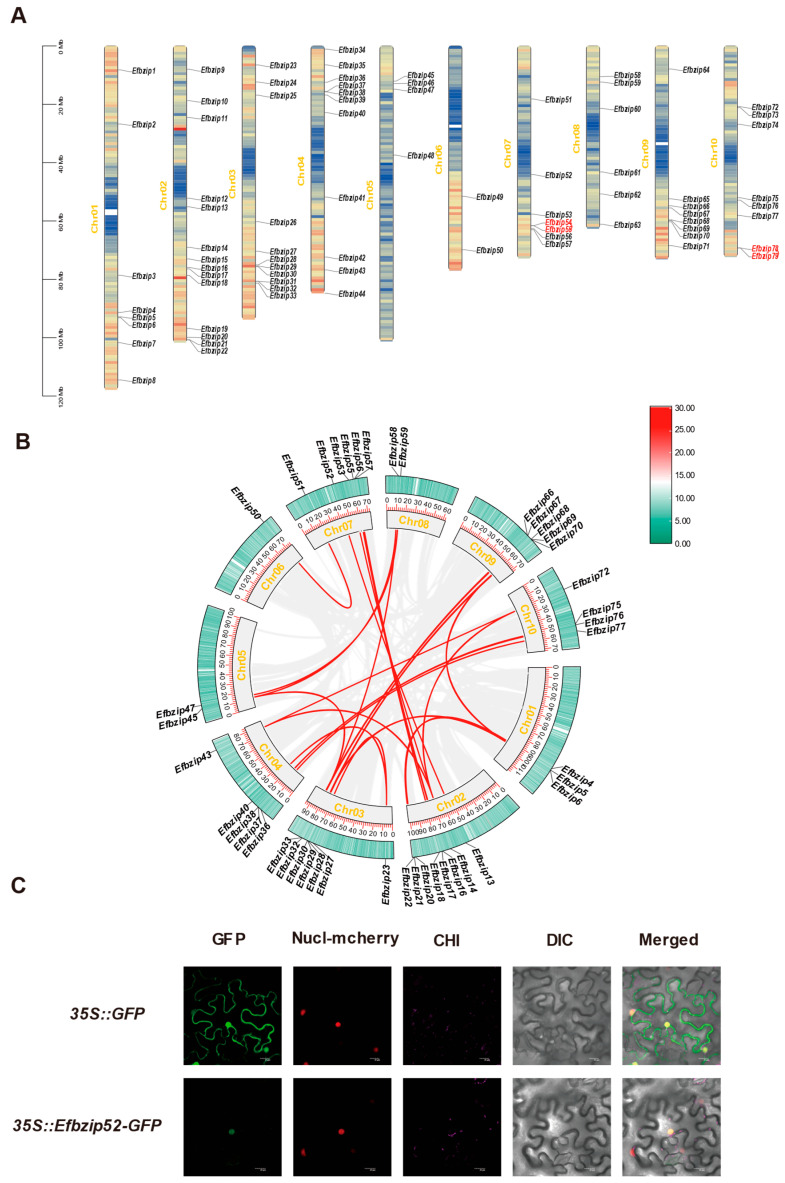
Chromosomal localization, intraspecific synteny, and subcellular localization analysis of *Efbzip* genes. (**A**) The chromosomal localization map of the *Efbzip* gene family members. (**B**) Intraspecific synteny analysis. (**C**) Subcellular localization of *Efbzip*52. The subcellular localization maps from left to right are GFP green fluorescent protein (GFP), nucleus marker (Nucl mcherry), chloroplast fluorescence channel (CHI), brightfield (DIC), and merged image. Gray lines indicate collinear genes within *E. fulvus*, while red highlights *Efbzip* collinear genes.

A genome-wide identification of bZIP gene family members in *E. fulvus* was performed using TBtools (V1.116), resulting in the identification of 79 genes. Based on chromosomal location, these genes were named *Efbzip1-79*. They were unevenly distributed across 10 chromosomes: Chr01 (8), Chr02 (14), Chr03 (12), Chr04 (11), Chr05 (4), Chr06 (2), Chr07 (7), Chr08 (6), Chr09 (8), and Chr10 (8) (Figure 1A). Physicochemical analysis revealed substantial variation among *Efbzip* proteins (Table S1): amino acid counts ranged from 144 to 669, and molecular weights ranged from 15,395.39 to 73,565.89 kDa, exhibiting consistent trends. Instability indices ranged from 40.33 to 82.75, indicating these proteins are unstable. Analysis of aliphatic indices showed values ranging between 39.49 and 93.99, while GRAVY scores varied from −1.042 to −0.122, indicating the hydrophilic nature of these proteins. The proteins exhibited a wide isoelectric point (pI) distribution, spanning from 4.48 to 10.96, with 38 proteins demonstrating a pI below 7, suggesting an absence of distinct acidic or alkaline predominance within this family. Predicted subcellular localization predominantly placed members within the nucleus, although exceptions were noted for *Efbzip*19 (endoplasmic reticulum), *Efbzip*48 (mitochondrion), *Efbzip*49 (chloroplast), *Efbzip*56 (peroxisome), *Efbzip*65 (endoplasmic reticulum), *Efbzip*78 (chloroplast), and *Efbzip*79 (cytoplasm). Experimental validation confirmed that *Efbzip*52 localized to the nucleus, consistent with the prediction (Figure 1C).

### 3.2. Gene Family Evolution Analysis

**Figure 2:**
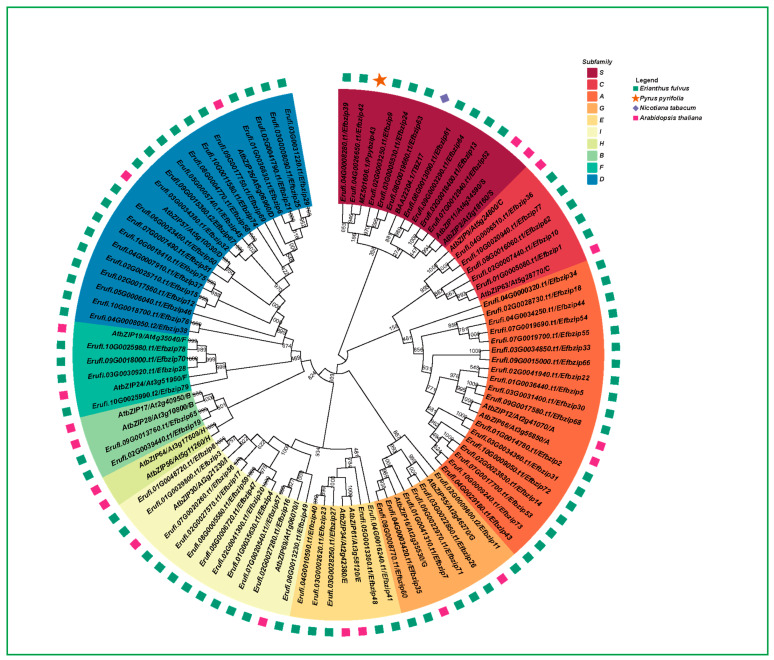
Evolutionary tree of the bZIP gene family (the bootstrap value is 1000).

To examine the phylogenetic relationships of the *E. fulvus Efbzip* gene family, the genes were classified into nine subfamilies using Arabidopsis thaliana as a reference (Figure 2). The number of members varied across subfamilies: A (18), B (2), C (5), D (19), E (5), F (4), G (6), I (11), and S (9). Members of the H subfamily in Arabidopsis did not cluster with *E. fulvus* bZIP genes and formed a separate branch. Within the *Efbzip* family, the A, D, and S subfamilies contained the most members, whereas the B subfamily had the fewest. These results indicate substantial variation in gene distribution among subfamilies.

### 3.3. Analysis of Promoter Cis-Acting Elements in Efbzip Gene Family Members

**Figure 3:**
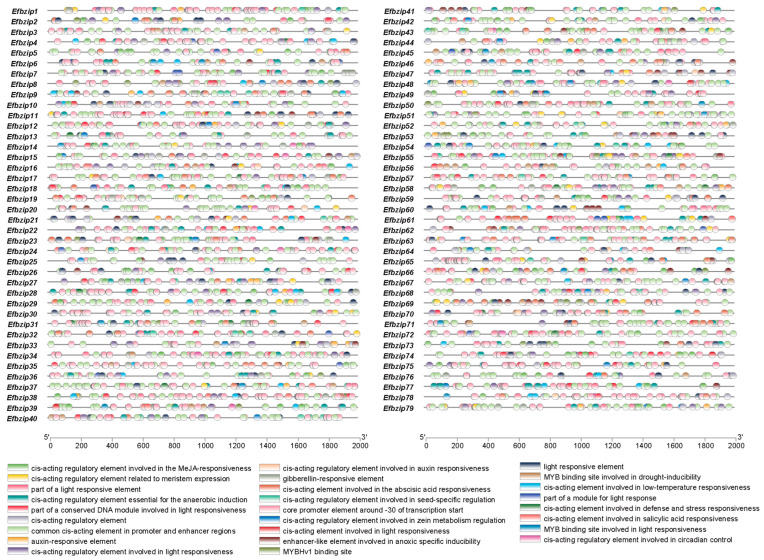
The cis-acting element functions in the promoter of Efbzip gene family members.

Analysis of the 2000 bp promoter regions from all 79 *Efbzip* genes identified 26 major cis-acting elements (Figure 3). These elements included those responsive to light, MeJA, abscisic acid, salicylic acid, cold, and anaerobic induction. Light-responsive elements were the most abundant (785 occurrences), followed by MeJA-responsive (332) and abscisic acid-responsive (261) elements (Table S3). These findings suggest that the *Efbzip* gene family may regulate sucrose metabolism through light signaling and hormone-mediated pathways.

### 3.4. Analysis of Gene Structure, Conserved Motifs, and Domains in Efbzip Family Members

**Figure 4:**
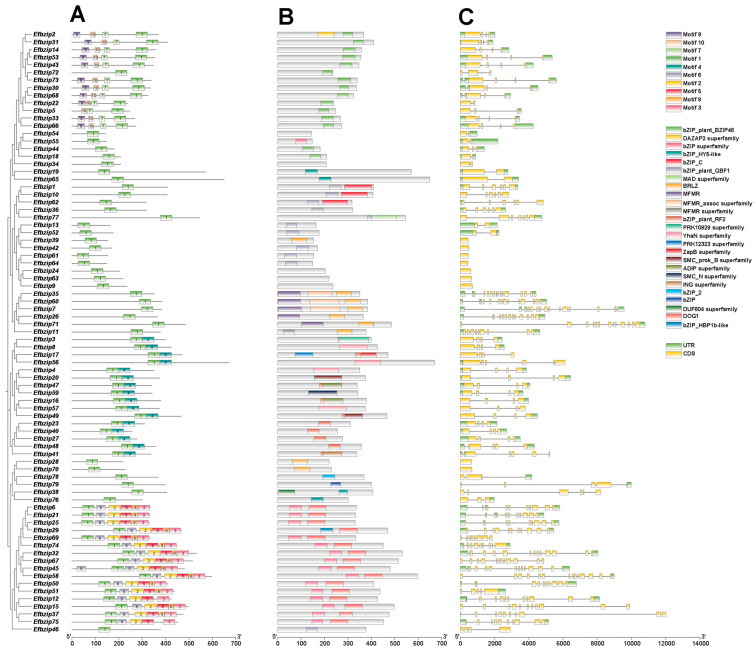
Sequence characteristics of *Efbzip* genes. (**A**) Conserved motifs; (**B**) Domains; (**C**) Gene structure.

Conserved motif prediction using TBtools (V1.116) identified 10 motifs (Motif 1–10) in *Efbzip* proteins (Figure 4A). All genes contained Motif 1. The A subfamily mainly contained Motif 7, Motif 9, and Motif 10; subfamilies E and I primarily contained Motif 4; and the D subfamily contained Motifs 2, 3, 5, 6, and 8. Domain analysis (Figure 4B) showed that: Subfamily A mainly contained the bZIP_plant_BZIP46 domain; Subfamilies S and C predominantly contained the bZIP_plant_GBF1 domain; Subfamily G primarily contained the MFMR and MFMR_assoc domains; Subfamily E mainly contained the bZIP_plant_RF2 domain; Subfamily B predominantly contained the bZIP_HY5-like domain; Subfamily D primarily contained the bZIP superfamily domain.

Gene structure analysis (Figure 4C) showed that members within the same subfamily had conserved gene structures. Seventeen genes lacked UTR sequences, whereas 78% of genes contained UTRs. These results indicate that gene structures are relatively conserved within subfamilies, while differences among subfamilies reflect functional diversification.

### 3.5. Synteny Analysis of the Efbzip Gene Family

Synteny analysis using TBtools (V1.116) identified 28 duplication events within the *Efbzip* family (Table S4), including 26 segmental duplications (Figure 1B) and 2 tandem duplications (Figure 1A). All chromosomes participated in segmental duplication events, while tandem duplications occurred only on Chr07 and Chr10. These results indicate that segmental duplication likely served as the primary driver of *Efbzip* gene family expansion in *E. fulvus*.

The divergence times of duplicated gene pairs ranged from 5.58 to 170 MYA, with 8 events within the past 60 MYA and 20 events occurring earlier. All Ka/Ks ratios were <1, suggesting that the duplicated genes experienced purifying selection.

**Figure 5:**
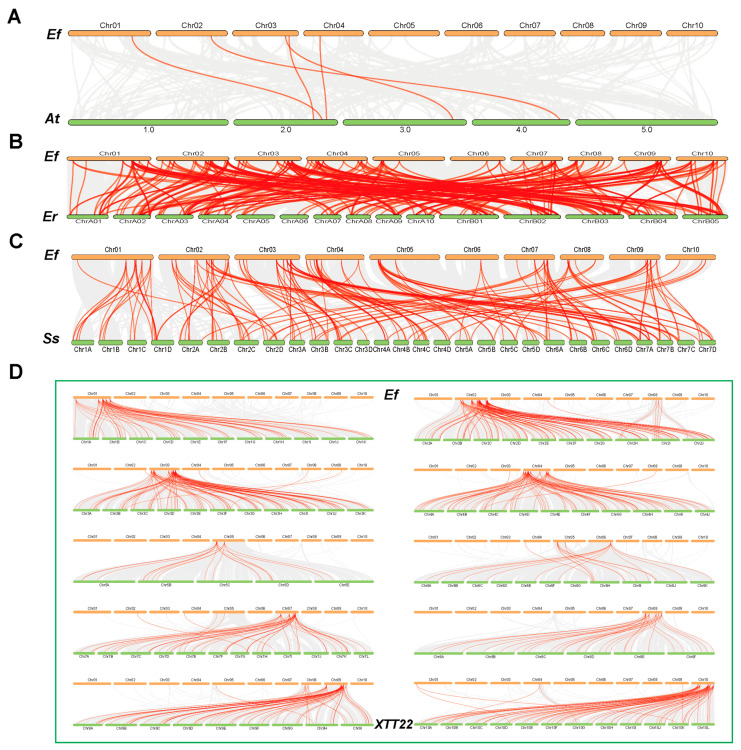
Interspecies collinearity analysis. (**A**) Collinearity analysis between Ef and At; (**B**) Collinearity analysis between Ef and Er; (**C**) Collinearity analysis between Ef and Ss; (**D**) Collinearity analysis between Ef and Ss. The gray line represents the collinearity genes between *E. fulvus* and other species, while the red line highlights the collinearity genes between *E. fulvus* Efbzip and other species.

To examine evolutionary relationships across species, interspecific synteny analysis was performed between *E. fulvus* (Ef) and *Arabidopsis thaliana* (At), *S. spontaneum* (Ss), *Erianthus rockii* (Er), and XTT22 (Figure 5). The numbers of collinear gene pairs (Table S5) were 5, 114, 262, and 597, respectively, indicating stronger synteny with species phylogenetically closer to *E. fulvus*.

### 3.6. Expression Patterns of the Efbzip Gene Family in Different Tissues

**Figure 6:**
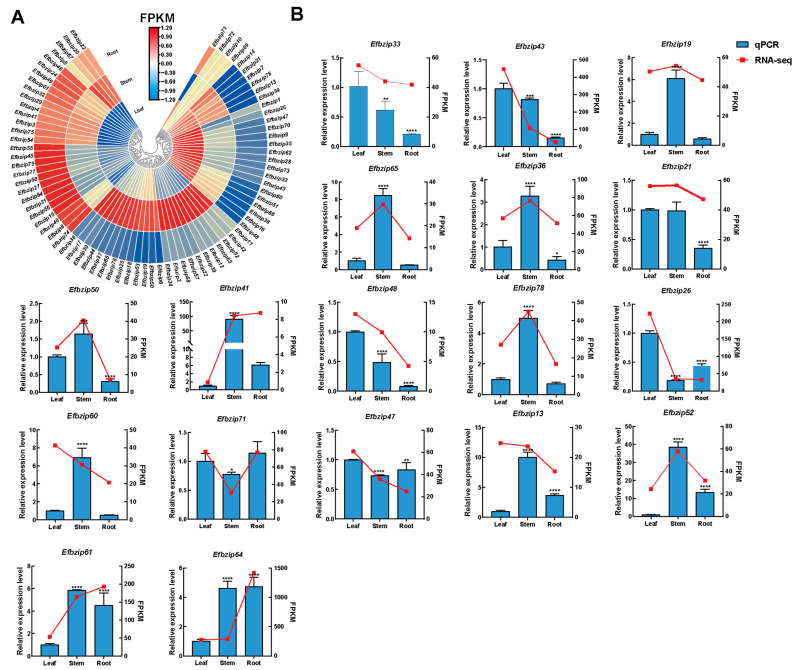
Tissue-specific expression patterns. (**A**) Transcriptome data heatmap. (**B**) Expression patterns of *Efbzip* genes in leaves, stems, and roots. Statistical significance is indicated as follows: * *p* < 0.05, ** *p* < 0.01, *** *p* < 0.001, **** *p* < 0.0001. Error bar: SD.

To further validate the tissue-specific expression characteristics of the *Efbzip* gene family, 18 genes showing high transcript levels from each subfamily were identified based on available transcriptomic data [42] (Figure 6A, Table S6). A combination of qRT-PCR assays and transcriptome analysis was utilized. Within subfamily A, *Efbzip33* and *Efbzip43* exhibited the greatest expression in leaves, notably higher than in stems and roots. For subfamily B, *Efbzip19* and *Efbzip65* were predominantly expressed in stems, significantly surpassing expression levels in leaves and roots. Subfamily C’s *Efbzip36* was chiefly expressed in stems, substantially higher compared to leaves and roots. In subfamily D, elevated leaf expression was noted for *Efbzip21* and *Efbzip50*, markedly greater than root expression levels. In subfamily E, *Efbzip41* and *Efbzip48* demonstrated peak expression in stems and leaves, respectively. *Efbzip78* from subfamily F presented highest stem expression, significantly exceeding that observed in leaves and roots. Within, *Efbzip60* had prominent expression in stems, markedly higher than in leaves and roots; *Efbzip71* and *Efbzip26* were predominantly expressed in leaves, significantly exceeding expression in stems and roots. In subfamily I, *Efbzip47* was most expressed in leaves, considerably higher than in stems and roots. Subfamily S members *Efbzip13*, *Efbzip52*, *Efbzip61*, and *Efbzip64* showed significantly elevated transcript levels in stems and roots compared to leaves. Overall, the *Efbzip* gene family in *E. fulvus* was mainly expressed in stems and leaves, with the fewest highly expressed genes found in roots. Members of the same subfamily generally exhibited similar expression patterns.

### 3.7. Identification of Genes Regulating Sucrose Metabolism Among Efbzip Family Members

**Figure 7:**
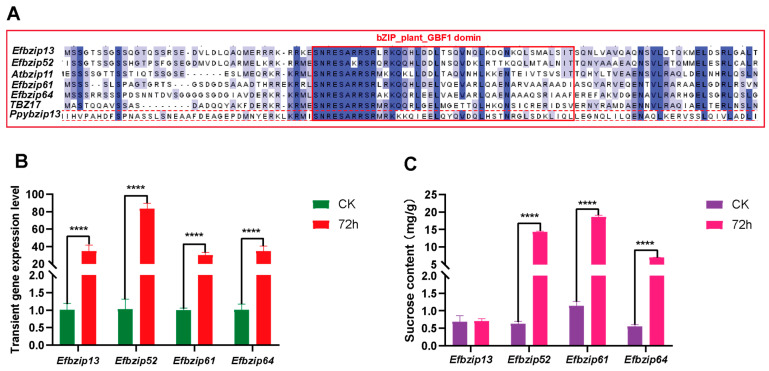
Analysis of transient expression impacts of *Efbzip* genes on sucrose metabolism. (**A**) Protein sequence alignment. (**B**) Quantification of gene expression. (**C**) Determination of sucrose content. Statistical significance was set as follows: **** *p* < 0.0001. Error bar: SD.

Previous studies suggest structurally similar genes may share functional similarities. To identify potential regulators of sucrose metabolism within the *Efbzip* family, a phylogenetic tree was constructed (Figure 3), including known sucrose-related bZIP genes (*Ppybzip43*, *TBZ17*, *Atbzip11*) from other species and *E. fulvus Efbzip* members. Protein sequence alignment was also visualized (Figure 7A). Phylogenetic analysis revealed *Efbzip13*, *Efbzip52*, *Efbzip61*, and *Efbzip64* clustered closely. These four genes underwent cloning, and overexpression vectors were subsequently generated using homologous recombination techniques. Nicotiana benthamiana plants were utilized for transient expression assays. Compared with the control (CK, Figure 7B), a marked increase in transcript abundance for all four genes was observed at 72 h post-transfection. Furthermore, the transient overexpression of *Efbzip52*, *Efbzip61*, and *Efbzip64* notably elevated sucrose levels in tobacco leaves (Figure 7C), while *Efbzip13* displayed no significant impact. These findings suggest that *Efbzip52*, *Efbzip61*, and *Efbzip64* may play crucial roles in sucrose metabolism within sugarcane, marking them as potential genetic targets for developing high-sugar cultivars.

## 4. Discussion

bZIP transcription factors are very important for the metabolism of sucrose in the plant, especially through the control of sugar signaling and expression of the related genes. From the *E. fulvus* genome, a total of 79 *EfbZIP* transcription factors were found, and there were differences observed in the number of bZIP genes found in other species. For example, *Oryza sativa* has (71) [7], *Setaria italica* (92) [13], *Salvia miltiorrhiza* (70) [14], *Andrographis paniculata* (62) [12], *Gossypium hirsutum* L. (207) [11], *Solanum tuberosum* L. (65) [8], *Ricinus communis* L. (49) [9], *Pyrus* spp. (84) [10], and *Arabidopsis* (75) [16]. These variations may result from species-specific evolutionary events or differences in genome size.

The instability indices of *EfbZIP* proteins, determined using physicochemical properties, vary between 40.33 and 82.75, which is low. Proteins with indices above 40 are believed to be unstable [44]. High instability is closely related to the structure and functions of the proteins, probably involving the dynamic biological functions of stress response and signal transduction [45].

Based on the information of the Arabidopsis bZIP gene family, the *E. fulvus* bZIP gene family consists of nine subfamilies (A, B, C, D, E, F, G, I, and S), each having a different number of genes. There are 18 genes belonging to subfamily A, while only two genes are found in subfamily B. These asymmetrical distributions might suggest expansions and contractions within the gene family of *E. fulvus* [46,47]. Tandem duplications and segmental duplications are common genetic events that occur within the *E. fulvus* genome [37]. The same is true for the bZIP gene family of the sugarcane plant, where the subfamilies that have a large number of genes (A and D, for instance) are believed to have undergone repeated gene duplication events, thus having important functions relating to environmental adaptations. Moreover, the genes of the H subfamily of the Arabidopsis are not related to any of the *E. fulvus* bZIP genes of the *EfbZIP* subgroup, having undergone elimination and having been replaced with other functions during the course of *E. fulvus* evolution [39]. Studies have also previously shown that the S subfamily of the Arabidopsis bZIP genes is involved in the metabolism of sugars such as sucrose [16]. We hypothesize that the S subfamily in *E. fulvus* may have similar functions, although further experimental validation is required.

Motif analysis indicates that each gene contains the same motif, a phenomenon also observed in rice [48]. Furthermore, motif diversity varies across subfamilies, potentially due to functional differentiation, a phenomenon previously reported in poplar [49]. Domain examination showed the presence of subfamily-specific domains. This condition has also been found among other species, such as the mangosteen plant and other organisms [50]. Gene structure analysis indicated relatively conserved gene structures among members of the same subfamily, consistent with findings in the bZIP gene family of *Lycium barbarum* [51]. In summary, the conservation within subfamilies and differences among subfamilies in *E. fulvus Efbzip* genes not only support the evolutionary conservation of the bZIP gene family across species but also demonstrate its functional diversification [52,53]. The bZIP transcription factor light response element dominates across multiple species, including *E. fulvus*, rice, and Arabidopsis [16,48,49,54,55], indicating its potential in regulating light signal transduction.

In an intraspecific collinearity analysis, 28 duplication events were observed, consisting of 26 segmental duplication pairs and 2 tandem duplication pairs. This indicates that gene duplication likely represents the primary mechanism underlying the *Efbzip* gene family’s expansion, consistent with patterns observed across other plant gene families [56,57,58,59,60]. The differentiation time of duplicated gene pairs ranges from 5.58 to 170 million years (MYA). Among these, 8 pairs emerged within 60 MYA, and 20 pairs appeared earlier than 60 MYA, indicating multiple gene expansion events over a long evolutionary period. Additionally, all duplicated events appear to have undergone purifying selection pressure, suggesting these genes maintain functional conservation essential for fundamental biological processes [61].

In interspecies comparisons, the high homology between Ef and XTT22 as well as Er reflects their close phylogenetic relationships and shared evolutionary histories. In contrast, Ef shares only five collinear gene pairs with At, indicating significant divergence in the evolution of their bZIP gene families. This divergence likely results from the phylogenetic distance between dicotyledons and monocotyledons. The 114 collinear gene pairs between Ef and Ss suggest partial conservation, possibly related to their shared ancestry within the Poaceae family. Collectively, these interspecies differences underscore the dynamic evolution of the bZIP gene family in plants: closely related species (e.g., Ef, Er, and XTT22) maintain high homology through gene duplication, while distantly related species exhibit more divergent gene family structures due to independent evolutionary events [62,63,64].

*Efbzip* genes display tissue-specific expression patterns consistent with previous studies [65,66]. Furthermore, existing research indicates that tissue-specific gene expression may correlate with the gene’s biological function [66,67,68,69]. Therefore, studying the gene’s expression patterns is important for further functional research.

Phylogenetic analysis revealed that *Efbzip13*, *Efbzip52*, *Efbzip61*, and *Efbzip64* clustered with known bZIP genes associated with sucrose metabolism (such as *PpybZIP43*, *TBZ17*, and *AtbZIP11*), supporting the hypothesis that structurally similar genes possess analogous functions [70]. Subsequent functional validation revealed that transient overexpression of *Efbzip52*, *Efbzip61*, and *Efbzip64* in Nicotiana benthamiana significantly increased the sucrose content in tobacco leaves, whereas *Efbzip13* did not exhibit this effect. This suggests that *Efbzip52*, *Efbzip61*, and *Efbzip64* may directly or indirectly regulate sucrose metabolism. These findings reinforce the utility of phylogenetic analysis in predicting gene functions and highlight the potential of *Efbzip52*, *Efbzip61*, and *Efbzip64* as sucrose metabolism regulators. The lack of effect observed with *Efbzip13* may result from structural fine-tuning (such as sequence variations outside conserved domains) affecting protein interaction specificity [71,72] or promoter evolution [73].

This study has some inherent limitations. Firstly, gene functions identified in model plants may not necessarily correspond to identical roles in different species. Secondly, there is currently insufficient experimental evidence validating the functions of these genes. To overcome these constraints, future investigations should incorporate advanced genetic methodologies such as overexpression and CRISPR-Cas9-mediated gene editing in both sugarcane and *E. fulvus*.

## 5. Conclusions

This study systematically identified a total of 79 *Efbzip* genes belonging to the *Efbzip* gene family in the *E. fulvus* genome, of which the genes are unequally distributed across the 10 chromosomes. Subcellular localization analysis predicted that the members of the *Efbzip* gene family are nuclear. Phylogenetic analysis classified the 79 identified *Efbzip* genes into nine subfamilies. The analysis of the structures of the *Efbzip* genes revealed the widespread distribution of Motif1 and the consensus of each subfamily. The analysis of the cis-active promoter elements further revealed that the *Efbzip* gene family is often involved in the light and hormone regulatory pathways of *E. fulvus*. The gene duplication event of the *Efbzip* gene family in *E. fulvus*, revealed through the phylogenetic analysis, included the segment and tandem duplication events of the *Efbzip* gene family. The expression of the *Efbzip* genes was further confirmed using the qRT-PCR analysis. The expression of the bZIP genes was largely observed in stems and leaves, with relatively low expression of specific genes in the roots. There was a trend of similar expression among members of the same subfamily. Transient overexpression of *Efbzip52*, *Efbzip61*, and *Efbzip64* led to significantly elevated sucrose content in tobacco leaves (*p* < 0.0001). Overall, this study represents the first extensive characterization of the bZIP transcription factor gene family in *E. fulvus* and identifies promising candidate genes for the genetic improvement of sucrose metabolism traits in sugarcane.

## Data Availability

Data supporting the results of this study are available from the corresponding authors upon reasonable request.

## Supplementary Materials

The following supporting information can be downloaded at https://www.mdpi.com/article/10.3390/genes16121434/s1, Table S1: Physicochemical properties analysis of 79 proteins; Table S2: The secondary structure of 79 proteins; Table S3: Cis-acting elements of the promoter region; Table S4: Segmentally and tandemly duplicated gene pairs; Table S5: The number of collinear genes between the E. fulvus Efbzip gene family and other different species; Table S6: RNA-seq data (FPKM values) of 79 genes; Table S7: Primer sequences for qRT-PCR and gene cloning.
